# Changes in utilization of health services among poor and rural residents in Uganda: are reforms benefitting the poor?

**DOI:** 10.1186/1475-9276-8-39

**Published:** 2009-11-12

**Authors:** George W Pariyo, Elizabeth Ekirapa-Kiracho, Olico Okui, Mohammed Hafizur Rahman, Stefan Peterson, David M Bishai, Henry Lucas, David H Peters

**Affiliations:** 1 Department of Health Policy, Planning and Management, Makerere University School of Public Health, P.O. Box 7072, Kampala, Uganda; 2 Department of International Health, Johns Hopkins Bloomberg School of Public Health, 615 North Wolfe Street, Baltimore, MD 21205, USA; 3 Division of International Health (IHCAR), Karolinska Institutet, S-171 77 Stockholm, Sweden; 4 Department of Population and Family Health Sciences, Johns Hopkins Bloomberg School of Public Health, 615 North Wolfe Street, Baltimore, MD 21205, USA; 5 Institute of Development Studies, at the University of Sussex, Brighton, BN1 9RE, UK

## Abstract

**Background:**

Uganda implemented health sector reforms to make services more accessible to the population. An assessment of the likely impact of these reforms is important for informing policy. This paper describes the changes in utilization of health services that occurred among the poor and those in rural areas between 2002/3 and 2005/6 and associated factors.

**Methods:**

Secondary data analysis was done using the socio-economic component of the Uganda National Household Surveys 2002/03 and 2005/06. The poor were identified from wealth quintiles constructed using an asset based index derived from Principal Components Analysis (PCA). The probability of choice of health care provider was assessed using multinomial logistic regression and multi-level statistical models.

**Results:**

The odds of not seeking care in 2005/6 were 1.79 times higher than in 2002/3 (OR = 1.79; 95% CI 1.65 - 1.94). The rural population experienced a 43% reduction in the risk of not seeking care because of poor geographical access (OR = 0.57; 95% CI 0.48 - 0.67). The risk of not seeking care due to high costs did not change significantly. Private for profit providers (PFP) were the major providers of services in 2002/3 and 2005/6. Using PFP as base category, respondents were more likely to have used private not for profit (PNFP) in 2005/6 than in 2002/3 (OR = 2.15; 95% CI 1.58 - 2.92), and also more likely to use public facilities in 2005/6 than 2002/3 (OR = 1.31; 95% CI 1.15 - 1.48). The most poor, females, rural residents, and those from elderly headed households were more likely to use public facilities relative to PFP.

**Conclusion:**

Although overall utilization of public and PNFP services by rural and poor populations had increased, PFP remained the major source of care. The odds of not seeking care due to distance decreased in rural areas but cost continued to be an important barrier to seeking health services for residents from poor, rural, and elderly headed households. Policy makers should consider targeting subsidies to the poor and rural populations. Public private partnerships should be broadened to increase access to health services among the vulnerable.

## Background

Several factors such as proximity to health care providers, perceived quality of care, fees charged and perceived severity of illness have been shown to affect access and utilization of health services [1-6].

Uganda implemented a number of health sector reforms in an attempt to improve access to health services. These included introduction and then abolition of user-fees, decentralization of responsibility for delivery of health services to local authorities, restructuring of Ministry of Health (MOH), introduction of the Uganda National Minimum Health Care Package (UNMHCP), and autonomy for the National Medical Stores (NMS). There were also various experiments with prepayment and community health insurance schemes, contracting with health workers, and hospital autonomy. Some of the main reasons in favour of reforms for the health sector included failure to make appreciable progress towards the primary health care (PHC) goals of equitable health care, fragmentation of the health sector, and inability of the MOH to take charge of the health sector through sound policy and legislation [7]. These reforms took place along with other changes in the public sector consisting of liberalization and privatization, constitutional reforms, civil service reforms, and broader decentralization efforts [8]. Some of these reforms, however, were not based on locally generated ideas, objective assessments of the existing situations, or local adaptation of interventions tried elsewhere, but on pre-packaged interventions designed by donor agencies [7]. In one response to reduce this problem and better harmonise resource inflows for planning in the health sector, budget support mechanisms were introduced with significant amounts of donor funding channelled through the national budget process in a sector wide approach (SWAp) [9,10].

The political and civil instability during the 1970's through to the mid 1980's greatly affected health service delivery in Uganda. There was stagnation of health policy formulation, infrastructure development, health service organization and delivery. Expansion of health service delivery to rural and underserved areas was virtually impossible because of the limited health budget and insecurity in some areas. With return of relative peace in the mid-1980s there was an influx of international humanitarian organizations. Some of these organizations initially provided relief services like food, first aid and emergency requirements for settlement, but eventually registered locally as Non Governmental Organizations (NGO), and became involved in providing or supporting health care delivery. In the absence of a national health policy, various stakeholders and projects led to the dominance of selective vertical health programs. In an attempt to address this situation, the Ugandan government embarked on a mission of rebuilding the health sector [7,11].

One of the early reforms was the introduction of user fees in the 1980's. It was hoped that this would result in improved quality of services and subsequently increase utilization. User-fees reforms require specific design elements, complementary government policies and contextual requirements for them to have positive efficiency and equity impacts [11]. This policy change was, however, implemented in a fragmented manner. Limited attention was paid to the design elements and contextual issues within the country, hence the policy did not result in the generation of significant additional funds or improved quality of services; instead there was reduced utilization of services [7]. This was accompanied by an outcry about inability by the poor to access services, and consequently the abolition of user fees in 2001, with resulting increase in utilization in public facilities [12,13]. However, catastrophic expenditure did not decrease among the poor [14].

Another major reform that influenced health service delivery was large-scale decentralization of governance to districts with devolution of powers to allocate resources and deliver services (including health care), which was initiated in the early 1990's. In Uganda, decentralization was initiated largely to achieve political objectives but not primarily as an instrument for reforming the health sector [15]. However, the objectives for introducing decentralization usually include improving planning and management through decision making that is more responsive to local needs, improving service organization by reducing duplication, increasing accountability and promoting popular participation to encourage self reliance [11]. Enabling frameworks, both policy and legal, were enacted in the country and it resulted in both positive and negative modifications in the organization of health services and policy formulation. On average physical access to health facilities increased from 49% (2001) to 72% (2004) of the population living within 5 km of a health facility. However, changes in the health status of the population did not improve significantly as evidenced by the high infant and maternal mortality rates [15-17].

Another reform involved the provision of subsidies to the private not for profit (PNFP) health facilities since 1997. In return these facilities were expected to reduce the amount of fees levied. Some facilities were able to reduce fees substantially whereas others did not [4,18]. The failure to reduce fees was attributed to the challenge of ever rising operational costs [19,20]. The PNFP's also argued that the subsidies from government only covered one third of the cost of providing care, the other two thirds being met from PNFP solicited external donors and user fees [17,21]. The fees charged thus continued to operate at PNFP facilities and policy debates concerning their impact on the poor and on the merits and demerits of providing subsidies to the PNFP sub-sector continued. This paper intends to assess the changes in utilization patterns that occurred over that time. The choice to use data collected in the surveys conducted during parts of the years 2002 and 2003 (2002/3) and 2005 and 2006 (2005/6) is because the period coincides well with the time when we would expect the reforms to have been actively implemented throughout the country as part of the Health Sector Strategic Plan Phase I which ran from the fiscal years 2000/1 to 2004/5. The effects of the reforms would be expected to be detectable. In this sense we have taken 2002/3 data to act as baseline. Earlier than 2002/3 would be before or too close to initiation of the reforms for effects to have been felt, given that policy reforms are likely to take time to be assimilated, implemented, and to produce results.

The poor are most vulnerable to changes in the delivery and financing of health services. Many of the various health sector reforms were instituted in an attempt to improve access to health services for the poor. An empirical assessment of the likely impact of these policies is therefore important for informing further policy decision making. This paper attempts to describe the changes in utilization of health services (both outpatient and inpatient), that occurred among the poor and those in rural areas between 2002/03 and 2005/06, and identify some of the factors associated with these changes.

## Methods

The Uganda National Household Survey (UNHS) is a nationally representative sample survey conducted periodically by the Uganda Bureau of Statistics (UBoS, http://www.ubos.org) to provide data for planning and to inform the national budget process. These surveys employ standard methods with generally comparable variables between rounds and maintaining continuity over rounds to enable pooling of results over rounds if ever considered necessary [16,22,23]. The surveys were based on stratified two stage sampling with over-sampling of urban areas, and of some rural areas with concentrated informal sector activity. Each district was a stratum and was divided into rural and urban sub-strata. The total number of about 1,000 primary sampling units (PSUs) was firstly allocated between urban and rural in the proportion of 40:60 in 2002/3 and 20:80 in 2005/6. Thereafter, the urban and rural samples were generally allocated between the strata in proportion to the number of households with certain adjustments. The allocated sample was selected with probability proportional to number of households. A suitable plan for sub-stratification and selection of households at the listing stage was introduced to ensure adequate representation of households with at least one unemployed person and an informal sector enterprise activity. Further details on the sampling strategies and weighting used are available on http://www.ubos.org[22]. The surveys included questions on health care utilization for all household members and age groups. Secondary data analysis of the UNHS 2002/3 [22,23] and 2005/6 [16] was carried out using STATA Version 10 [24].

Univariate, bivariate and multivariable analysis was done. The poor were identified in the UNHS datasets based on membership to households assigned to one of five possible wealth categories (quintiles). The quintiles were generated from an asset index derived using Principal Components Analysis (PCA) and scoring of the first principal component [25]. The same assets were used in 2002/3 and 2005/6. The assets used included the material used for construction of the roof, wall, and floor of dwelling house, fuel used for lighting and cooking, the type of toilet, possession of a television set, mobile phone and radio. The choice of an asset-based index was driven by the desire to conform to other studies in Uganda and elsewhere, and for ease of data collection of subsequent facility and household surveys. The weights and cut offs were done separately for each survey, but they were based on the same items. Those individuals identified as coming from the bottom wealth quintile were defined as "most poor" for purposes of this analysis. The probability that a household member sought care for illness from public, private not for profit, or private for profit was modelled using Stata's generalised linear latent and mixed models (GLLAMM) feature [24], specifying the multinomial logit link. This choice was considered to be more appropriate as the outcome (choice of health provider) is of non-binary; categorical nature and the model accounts for clustering effects. The multi-level models were used to account for variables that operated at a household level and some that operated at a district level. The district is the administrative and geographical unit for local authority governance and service provision. The outcome variable of interest was choice of service provider for use of health services for an illness in the last 30 days. The surveys collected data on use of public (government) facilities, private not for profit (PNFP), and private clinic/drug shop, referred to in this paper as private for profit (PFP). The model uses attendance at PFP as the comparison category. Those who chose not to seek care, although of interest, were excluded from the model as data was not collected separately on them in the 2005/6 survey. We modelled the effects on the outcome of female gender, rural residence, distance to facility, household headship by vulnerable persons (females and elderly), household wealth quintile and passage of time between the two surveys (2005/6 = 1). Data on costs of seeking care was not readily identifiable or consistent between the two surveys.

## Results

### Sample characteristics

The study sample consisted of 52,088 individuals drawn from 9,711 households in the 2002/3 survey and 42,227 individuals from 7,426 households in 2005/6 surveys respectively (Table 1). The survey data were weighted accordingly to enable pooling of results over rounds [22]. Respondents from rural households made up 85.3% and 84.2% of the samples in the 2002/3 and 2005/6 surveys respectively. Respondents from households categorised as most poor (quintile 1) formed 27.4% and 17.7% of the samples in the 2002/3 and 2005/6 surveys respectively.

**Table 1:** The Socio-demographic Characteristics of Poor and Rural Residents in the UNHS 2002/3 and 2005/6 surveys

Category	2002/3		2005/6	
**Sex**	**Frequency**	**Percent**	**Frequency**	**Percent**

Male	24,567	47.1	20,689	49.0

Female	25,945	49.9	20,594	48.8

Missing	1,575	3.0	942	2.2

Total	52,088	100.0	42,227	100.0

Residence*				

Rural	44,447	85.3	35,537	84.2

Urban	7,641	14.7	6,689	15.8

Total	52,088	100.0	42,227	100.0

**Wealth Quintiles¶**				

Quintile 1	13,572	27.4	7,060	17.7

Quintile 2	12,680	25.5	8,115	20.3

Quintile 3	9,759	19.6	8,682	21.7

Quintile 4	7,561	15.2	8,272	20.7

Quintile 5	5,982	12.3	7,818	19.6

Total	49,557	100.0	39,955	100.0

*These are weighted results as different strata compositions were used in 2002/3 and 2005/6.

¶The same items were used in 2002/3 and 2005/6 but asset weights and cut-offs were done separately for each survey. Unequal quintiles are due to lumping. An asset index was not constructed for those who had missing asset variables.

UNHS = Uganda National Household Survey.

In these samples, 28.3% of respondents in 2002/3 and 39.5% in 2005/6 reported an illness in the 30 days prior to the survey.

### Care-seeking for illness

In order to better understand why people seek or do not seek care when sick, analysis was done among those reporting illness, on the reasons for not seeking care. Some 1,019 out of 13,917 respondents (7.3%) and 1,912 out of 15,426 respondents (12.4%) reported that they had not sought care at all in 2002/3 and 2005/6 respectively, resulting in the odds of not seeking care in 2005/6 being 1.79 times higher than in 2002/3 (OR = 1.79; 95% CI 1.65 - 1.94) [see Additional file 1]. Care seeking may be influenced by perceived severity of illness. Among those reporting any illness at all in the 30 days prior to the survey, 38.8% and 45.8% said the illness was mild in 2002/3 and 2005/6 surveys respectively. However, it was not possible to assess the influence of perceived severity on utilization. Although there was a change for the better in rural areas, geographical access was found not to be a major reason for not seeking care. Long distance as a reason for not seeking care was mentioned by only 0.5% (n = 35,573) in 2005/6 among the rural population, down from 1.0% (n = 44,447) found in 2002/3 (OR = 0.57; 95% 95% CI 0.48 - 0.67). Among the most poor, there was no significant change in distance as a reason for not seeking care in the 2005/6 survey (1.0%, n = 7,061) from the 1.2% (n = 13,572) found in 2002/3 (OR = 0.87; 95% CI 0.65 - 1.15). The reason for not seeking care due to high costs did not change among the most poor in the 2005/6 survey (2.0%, n = 7,061) from the 2002/3 survey (2.0%, n = 13,572) (OR = 0.99; 95% CI 0.80 - 1.23). Similarly, among the rural dwellers, there was no significant change in high cost as a reason for not seeking care in 2005/6 (1.6%, n = 35,537) compared to 2002/3 (1.8%, n = 44,447) (OR = 0.89; 95% CI 0.80 - 1.00).

**Figure 1:**
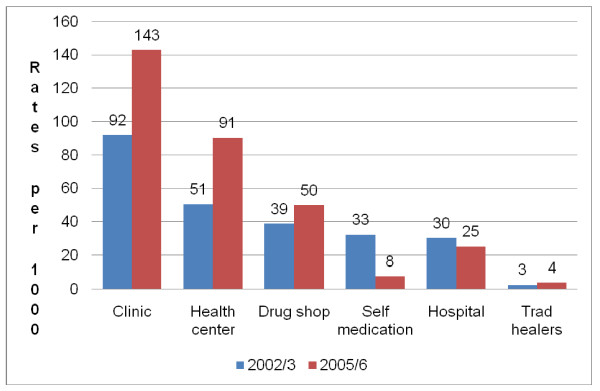
**Sources of care among the rural residents (UNHS 2002/3 and 2005/6)**. Chart showing health care utilisation rates for respondents in rural households by source of care based on secondary data analysis of the Uganda National Household Survey data from two surveys in 2002/3 and 2005/6.

**Figure 2:**
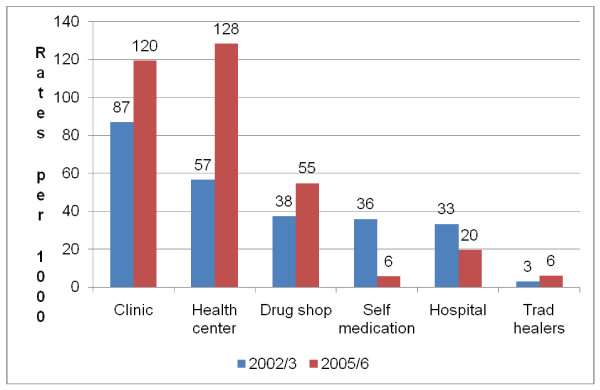
**Sources of care among the most poor residents (UNHS 2002/03 and 2005/06)**. Chart showing health care utilisation rates for respondents in the most poor households by source of care based on secondary data analysis of the Uganda National Household Survey data from two surveys in 2002/3 and 2005/6.

The remainder of the analysis focuses on the type of care utilized for those reporting an illness. The majority of the rural residents and most poor reported having utilised private clinics, health centres and drug shops in descending order in both 2002/3 and 2005/6 periods respectively (Figures 1 and 2).

Figures 1 and 2 show a similar pattern of utilization for both the rural and most poor. There were increases in utilization of clinics, health centres and drug shops while showing a decrease in self-medication and use of hospitals for those in rural areas as well as the most poor. There was an increase in utilization rates for health centres from 57 per 1,000 (95% CI 53 - 61) in 2002/3 to 128 per 1,000 (95% CI 121 - 136) in 2005/6 among the most poor. Similarly, there was an increase in utilization rates for health centres from 51 per 1,000 (95% CI 49 - 53) in 2002/3 to 91 per 1,000 (95% CI 88 - 93) in 2005/6. There was a significant reduction in the reported use of self medication from 33 per 1000 (95% CI 31 - 34) in 2002/3 to 8 per 1000 (95% CI 7 - 9) in 2005/6 among the rural residents. Similarly, there was a significant reduction in the reported use of self medication from 36 per 1000 (95% CI 33 - 39) in 2002/3 to 6 per 1000 (95% CI 4 - 8) in 2005/6 among the most poor residents. The reported use of traditional healers was negligible in both 2002/3 and 2005/6.

### Choice of provider

The probability of choice of care from PNFP or public facilities was first modelled using multinomial logistic regression, setting PFP as the base category, and accounting for household level cluster effects using Stata's robust cluster feature. We tested for the effects on the outcome of being female, rural residence, distance to facility, household headship by vulnerable persons (females and elderly), household wealth quintile and passage of time between the two surveys (2005/6 = 1). Respondents were more likely to have used PNFP in 2005/6 than in 2002/3 (RRR = 2.33; CI 1.75 - 3.12). Similarly respondents were more likely to have used public facilities in 2005/6 than in 2002/3 (RRR = 1.38; CI 1.13 - 1.50). Apart from passage of time there was no other significant predictor of choice of PNFP in this model. However, being female and membership to elderly headed households (60+ years) were associated with higher probability of using public facilities. There appears to be a socio-economic gradient with belonging to a higher wealth quintile being progressively associated with decreasing use of public facilities. This socio-economic gradient was not observed for utilization of PNFP facilities relative to PFP facilities (Table 2).

**Table 2:** Relative risk ratios in Multinomial Logistic Regression modelling for source of care (base category = attendance at private for profit; Data source - UNHS 2002/3 and 2005/6)

No of Level obs = 4285					
***Prob > chi2 = 0.0000***					

	**RRR**	**SE**	**P value**	**95% Confidence Intervals**	

**Private not for profit facility relative to Private for profit**					

Female	1.2214	.3083	.428	.7447	2.0034

Rural residence	1.1760	.2450	.436	.7817	1.7691

Within 5 km to health facility	.9935	.1404	.963	.753	1.3106

Vulnerable female headed Household	.7629	.2336	.377	.41866	1.3903

Vulnerable elderly headed HH	1.2813	.2407	.187	.8866	1.8517

Quintile 2	.6643	.1422	.056	.43672	1.0106

Quintile 3	.8888	.1759	.552	.603	1.3101

Quintile 4	.7790	.1599	.224	.5209	1.1651

Quintile 5	.7007	.1635	.128	.4435	1.1072

Time (2005/6 = 1)*	2.3398	.3435	.000	1.7546	3.1201

**Public facility relative to private for profit**					

					

Female*	1.3297	.1714	.027	1.0328	1.7119

Rural residence	1.0776	.1148	.483	0.8744	1.3281

Within 5 km from the health facility	.9667	.0689	.635	0.8405	1.1118

Vulnerable female headed household	1.1471	.1765	.372	0.8484	1.5510

Vulnerable elderly headed HH*	1.4891	.1439	.000	1.2321	1.7996

Quintile 2*	.7666	.0766	.008	0.6302	0.9324

Quintile 3*	.6860	.0692	.000	0.5629	0.8360

Quintile 4*	.5395	.0572	.000	0.4381	0.6642

Quintile 5*	.3837	.0478	.000	0.3005	0.4899

Time; (2005/6 = 1)*	1.3076	.0945	.000	1.1349	1.5066

(Private for profit is the base outcome) *Statistically significant.

UNHS = Uganda National Household Survey; RRR = Relative risk ratio; SE = Standard error

Considering that data was collected from a total of 56 districts in the two surveys, multi-level modelling using generalised linear, latent and mixed models (GLLAMM) was done to adjust for the clustering effects at household and district levels. The findings were generally similar to what was observed with the multinomial logistic regression, though a significant district level effect on the use of public facilities is shown in the multi-level model. Accounting for clustering produces a final model where rural residence becomes significant and where belonging to quintiles 2 and 3 are no longer significant (Table 3). The passage of time remained a significant predictor for use of both PNFP (OR = 2.15; CI 1.58 - 2.92), and public facilities (OR 1.31; CI 1.15 - 1.48). There was no other significant predictor in the model for use of PNFP. Being female remained significant for use of public facilities. When clustering by household and district are taken into account in the multi-level model, rural residence is found to be a significant predictor for use of public facilities. Being female, belonging to a household headed by elderly, and households in the 4^th ^and 5^th ^quintile remained significant predictors for use of public facilities (Table 3).

**Table 3:** Odds ratios in multilevel modelling for source of care (GLLAMM) (base category = attendance at private for profit; Data source - UNHS 2002/3 and 2005/6)

No of Level 1 units = 2806					
No of level 2 units = 56					
	**OR**	**SE**	**P value**	**Confidence interval**	

**PNFP relative to private for profit**					

Female	1.2245	.3207	.439	.7327	2.0462

Rural residence	1.4229	.3237	.121	.9109	2.2226

Within 5 km to health facility	.9954	.1477	.976	.7442	1.3314

Vulnerable female headed Household	.7287	.2319	.320	.3905	1.3597

Vulnerable elderly headed HH	1.3924	.2742	.093	.9466	2.0483

Quintile 2	.6735	.1539	.084	.4303	1.0541

Quintile 3	.9459	.2036	.796	.6203	1.4425

Quintile 4	.8714	.1973	.543	.5590	1.3584

Quintile 5	.7930	.2087	.379	.4733	1.3286

Time (2005/6 = 1)*	2.1485	.3348	.000	1.583	2.9160

Variances and covariances of random effects level 2 (districts) var(1): 6468 (.2091) Confidence interval = (0.22-1.06)					

**Public facility relative to private for profit**					

Female*	1.3773	1.5008	.003	1.1124	1.7052

Rural residence*	1.3176	.1221	.003	1.0986	1.5801

Within 5 km from the health facility	.9301	.0576	.242	.8238	1.0501

Vulnerable female headed household	1.084	.1428	.539	.8375	1.4039

Vulnerable elderly headed HH*	1.5383	.1316	.000	1.3007	1.8193

Quintile 2	.9287	.0860	.425	.7746	1.1136

Quintile 3	.8957	.0852	.247	.7433	1.0793

Quintile 4*	.7470	.0745	.003	.6143	.9085

Quintile 5*	.5479	.0630	.000	.4373	.6865

Time (2005/6 = 1)*	1.3092	.0837	.000	1.1549	1.4841

Variances and covariance's of random effects level 2 (districts) var(1): .4277 (.1030) Confidence interval = 0.2217,0.6338					

*Statistically significant.

UNHS = Uganda National Household Survey; GLLAMM = Generalized linear latent and mixed models; OR = Odds ratio; SE = Standard error

The choice of health care may also be influenced by level of severity of the condition. Unfortunately the UNHS data does not include variables on severity of the condition and we were unable to find a suitable proxy for severity.

## Discussion

In this section we focus the discussion on the changes that took place in sources of health care between 2002/3 and 2005/6 and on the implications of these changes for the poor and those who reside in the rural areas.

The odds of not seeking care increased in 2005/6 compared to 2002/3. In both surveys, one of the most frequent reasons given for not seeking care among those who did not consider their sickness mild was the high cost of seeking care. The influence of cost could not be tested using multi-level modelling because data on costs of seeking care were not readily identifiable or consistent between the two surveys.

However, studies done elsewhere have indicated that cost is often a barrier to seeking services especially for the poor [1,4,26,27]. Investment in health services by the government remains low and falls below the estimated minimum to provide the basic health care package [28]. This has resulted in gaps in service delivery such as lack of fully functional laboratories, stock-outs of medicines and supplies, and inadequately skilled, under-supervised and poorly motivated health workers. These gaps have resulted in use of private drug shops, pharmacies and laboratories even when consultation could be provided in the public facilities. This could also explain use of private clinics amidst "free" care in public facilities.

Another reason that was given for not seeking care was poor geographical access to health facilities. Although citing distance as a reason for not seeking care decreased by 43% among the rural residents there was no significant decline among the most poor. Not all rural residents are poor and distance as a barrier may not be perceived to the same degree by the poor and less poor. It is possible that further analysis may reveal that a majority of respondents who did not report distance as a barrier in rural areas may belong to less poor households, able to pay for transportation to far off facilities while the poor in rural areas cannot afford transport. Furthermore, those who chose not to seek care may have done so out of concern over costs of seeking care rather than severity of illness. Because of the lack of data on costs of health services, we were not able to assess this. On average, physical access, measured as the population living within 5 km of a health facility, increased from 49% in 1999 to 72% in 2004 [29,30]. It is important to note that there is substantial variation in physical access [17,30]. Although distance was not significant in multi-variable analysis as a predictor for actual reported utilisation, the common mention of distance as a barrier to seeking care may suggest that health facilities are still perceived, especially by the most poor, to be too far for them to reach easily. Studies done elsewhere have also indicated that distance from a public health facility reduces poor people's likelihood of accessing care [31-33]. We know that in several of the rural areas where majority of the poor live, facilities have been put up but are not very functional, due to the absences of health workers or medicines, and inadequate budgets to operate the new facilities. Given this scenario, a respondent may consider a health facility as not being there, which could influence responses.

The majority of the respondents who fell sick 30 days preceding the survey sought care from private clinics. It is possible that the increased use of clinics and health centres may be related to the reported increase in illness incidence from 28.3% to 39.5% between 2002/3 and 2005/6. However, a similar picture of increased use of clinics has been found in other developing countries [3,5,34,35]. These surveys did not include information on the quality of services provided in the clinics. A study done in Tanzania indicated that even when they access services, the poor, the less educated, and the rural women were less likely to receive key ANC interventions [27]. Limited research has been done in the private for profit sector in Uganda. However, available evidence indicates that the sector is still largely unregulated and concerns have been raised about the training of the health workers, and the quality of care provided in these health facilities, as well as in public facilities [17,36,37]. Similar concerns about the quality of the services provided by the PFP sector have been raised in other low income countries [5,33].

Respondents were more likely to use PNFP and public facilities relative to PFP in 2005/6 than in 2002/3; more likely to use public if female or rural; and less likely to use public if less poor. It is plausible that this resulted from improved proximity to the health facilities, stemming from the decentralization policy, coupled with increased funding from debt relief, which resulted in the construction of more health centres. Indeed there was an increase in the utilisation of health centres especially among the most poor and the rural residents over the period. Many PNFP providers responded to subsidies to increase accessibility to services by the poor. These actions included reducing charges, flattening of fees, or even completely removing fees [4,38]. Although this increased accessibility of services for the poor, in some cases it reduced the revenue base of the PNFP facilities. The costs of production (especially staff salaries) continued to rise and the subsidies that they received from government and contributions from their donors did not increase proportionately [19,20].

The less poor (quintile 4) and least poor (quintile 5) were less likely to use government clinics relative to private clinics. This is expected because although government services are nominally free, there remain numerous problems related to the shortage of health workers, drugs, supplies and equipment, and so many of those who can afford to pay for better quality services go to the private sector [1,26]. This socioeconomic gradient was not observed in the utilization of PNFP services as was the case with public facilities relative to PFP services. This could have resulted from the PNFP sub-sector, unlike the private sub-sector, making a deliberate effort to keep their fees affordable even to the lower socioeconomic groups. Secondly, it is possible that the technical quality of services offered by PNFP was better than what the PFP and public facilities offered. If this was the case they may have tended to attract the better off users as well. These two effects would tend to cancel each other reducing the possibility of having a socioeconomic gradient. A previous study in Uganda that compared health care outputs between public and PNFP showed that some other factor seems to be at work in PNFP facilities [2].

Overall, the use of traditional healers was negligible. These findings are consistent with other studies [26,34]. However, this information could be under reported because of the stigma associated with their use.

Finally we would like to highlight some methodological and other considerations and how they might affect interpretation of the findings. In general secondary data analyses are limited by the fact that the objectives of the secondary analysis and the original surveys may not be well aligned. For instance, the cost of seeking care could not be modelled because of inconsistencies in the way cost information was captured. Severity of illness was also not modelled because this information was not available.

Given the design of the study, i.e., using two cross-sectional sample surveys, it is not possible to definitively relate the changes to the reforms. The allocation of primary sampling units between urban and rural used in the two surveys differed. However, proportionate allocation of households and the adjustments using weights [22] appear to have been adequate as this resulted in roughly equal percentages of rural households between the two surveys of 85.3% and 84.2% in the 2002/3 and 2005/6 surveys respectively. The two surveys differed in the composition of households belonging to different wealth quintiles. The quintiles are not equal, especially in 2002/3, due to lumping. In addition, although using the same items, the asset weights and cut-offs were done separately for each survey. The 2005/6 survey reported that a significant decline in poverty was observed in rural areas between 2002/3 and 2005/6 from 42.7% to 34.2% [16]. Possible differences in illness incidence between the most poor and least poor might affect the results particularly where comparisons are made between 2002/3 and 2005/6 for rural residents.

We would also like to highlight the importance of taking cluster effects at different levels into consideration during analysis for surveys such as the UNHS. For instance, when we did multivariable analysis accounting for the effect of districts (GLLAMM), rural residence emerged as having a significant effect on use of public facilities, whereas it was not significant when we did not account for the district effects (Tables 2 and 3).

## Conclusion

This analysis has shown that reforms that were implemented between 2002/3 and 2005/6 were associated with mixed results in terms of increased access for the poor. Overall utilization of public and PNFP services by rural and poor populations increased. Private for profit (PFP) continued to be the major providers of care in spite of the presence of "free" government services. This may subject sections of the population to high out of pocket expenditures, raising concerns especially for the poor. There is need to better influence the markets involving private for profit providers, given that they remain a major source of health care for the population including the poor. Innovative ways to ensure that care provided by these providers is safe and effective are needed, such as through better measurement of performance and accountability mechanisms, contracting, or engagement of consumer groups, provider associations and franchises [39].

The observation that government services started to take a larger share of utilisation, especially for the most poor, is encouraging if it means they receive better quality of care and reduce impoverishing health care expenditures, and suggests that the reforms may indeed have benefitted those for whom they were intended. However, the odds of not seeking care also increased, and financial access to services remained a problem for the most poor and rural populations while geographical access continued to be perceived to be a problem for the most poor. Governments and health development partners need to come up with innovative solutions in order to address the special health care needs of the poor.

Some of the main reasons that respondents gave for not seeking care included costs and long distance to health facilities, with the poorest quintile of the population and those in rural areas being the most affected. Targeted subsidies that offer more resources to providers who fill in a critical niche in the rural and hard to reach areas should be considered and evaluated. In addition, subsidies could also target the users of the health services, specifically the poor and vulnerable. Alternative financing mechanisms like community based health insurance could also be explored.

## Competing interests

The authors declare that they have no competing interests.

## Authors' contributions

GWP is the Ugandan PI for the Future Health Systems study team. GWP, EEK and OO initiated the concept for the secondary data analysis. GWP came up with analysis plan and supervised the data analysis, and co-wrote drafts of the manuscripts as well as being principal author for the final version of the manuscript. EEK performed the analysis reported in this paper and co-wrote drafts of the manuscript. OO contributed to formulation of the study questions and concept and co-wrote the manuscript. RH provided significant help with multi-level modelling. SP, DB, HL, and DHP all contributed to the formulation of the study, reviewed and provided substantial inputs into the manuscript. All authors read, provided substantial input and approved the final manuscript. GWP and EEK are guarantors of the paper.

## Supplementary Material

Additional file 1 **Appendix for the paper by Pariyo, Ekirapa-Kiracho et al Changes in utilisation among the rural and poor in Uganda**. This is an Excel file that contains two by two tables used in the calculation of odds ratios reported in the paper. Click here for file
